# CP-154,526 Modifies CREB Phosphorylation and Thioredoxin-1 Expression in the Dentate Gyrus following Morphine-Induced Conditioned Place Preference

**DOI:** 10.1371/journal.pone.0136164

**Published:** 2015-08-27

**Authors:** Juan-Antonio García-Carmona, Daymi M. Camejo, Pilar Almela, Ana Jiménez, María-Victoria Milanés, Francisca Sevilla, María-Luisa Laorden

**Affiliations:** 1 Department of Pharmacology, Faculty of Medicine, University of Murcia, Murcia, Spain; 2 Department of Stress Biology and Plant Pathology, CEBAS-CSIC, Murcia, Spain; Peking University, CHINA

## Abstract

Corticotropin-releasing factor (CRF) acts as neuro-regulator of the behavioral and emotional integration of environmental and endogenous stimuli associated with drug dependence. Thioredoxin-1 (Trx-1) is a functional protein controlling the redox status of several proteins, which is involved in addictive processes. In the present study, we have evaluated the role of CRF1 receptor (CRF1R) in the rewarding properties of morphine by using the conditioned place preference (CPP) paradigm. We also investigate the effects of the CRF1R antagonist, CP-154,526, on the morphine CPP-induced activation of CRF neurons, CREB phosphorylation and Trx expression in paraventricular nucleus (PVN) and dentate gyrus (DG) of the mice brain. CP-154,526 abolished the acquisition of morphine CPP and the increase of CRF/pCREB positive neurons in PVN. Moreover, this CRF1R antagonist prevented morphine-induced CRF-immunoreactive fibers in DG, as well as the increase in pCREB expression in both the PVN and DG. In addition, morphine exposure induced an increase in Trx-1 expression in DG without any alterations in PVN. We also observed that the majority of pCREB positive neurons in DG co-expressed Trx-1, suggesting that Trx-1 could activate CREB in the DG, a brain region involved in memory consolidation. Altogether, these results support the idea that CRF1R antagonist blocked Trx-1 expression and pCREB/Trx-1 co-localization, indicating a critical role of CRF, through CRF1R, in molecular changes involved in morphine associated behaviors.

## Introduction

The conditioned place preference (CPP) paradigm has been used extensively to investigate the motivational effects of drugs of abuse. Drugs of abuse act as reinforce because they influence learning and memory processes [1]. Hippocampus is a brain region having a key role in the modulation of associative processes, such as declarative memory [2]. A functional association between ventral tegmental area (VTA) and hippocampus has been suggested to link memory and rewarding centers of the brain [3]. Moreover, there is evidence showing that the hippocampus is involved in several rodent learning tasks, such as the CPP [4,5]. In fact, hippocampus plays an important role in the formation of contextual memory between the environmental and the rewarding effect of drugs of abuse [6].

Brain stress system has been also implicated in the regulation of reinforcing properties of drugs [7,8] and drug-associated cues [9,10]. Corticotropin-releasing factor (CRF) is an important mediator of stress responses both in hypothalamic and extrahypothalamic systems. With respect to hypothalamus, CRF release from paraventricular nucleus (PVN) controls the hypothalamic-pituitary-adrenal (HPA) axis responses to stress and drug addiction [11–13]. PVN has direct connections with dopaminergic neurons located in VTA projecting to nucleus accumbens (NAc) [14,15]. Given the relationship of PVN and hippocampus with mesolimbic pathways and the presence of CRF neurons in PVN and CRF fibers in dentate gyrus (DG), the effects of morphine CPP in both brain areas were assessed in the present study.

At the extrahypothalamic level, CRF acts as a neuro-regulator of the behavioral and emotional integration of environmental and endogenous stimuli associated with drug dependence [16,17]. CRF and its CRF1 receptor (CRF1R) are distributed widely and in a highly conserved way in several brain regions, including the hippocampal formation [18–20]. In the DG, a hippocampal area participating in the storage of past experiences and contexts [21], CRF release is triggered from inhibitory interneurons [22] through CRF1R [18] by environmental stimulus. The activation of CRF1R stimulates the Gαs protein leading to activation of protein kinase A, and the transcription factor cAMP response element binding protein (CREB) [23]. CREB-mediated transcription is thought to be critical for learning and memory, and it has been implicated in opioid addiction [24–26]. Previous studies suggest that the phosphorylation site of CREB is a convergence point for multiple kinases and acts as a molecular switch for controlling gene activation kinetics. CREB can also be activated by redox proteins as Thioredoxin-1 (Trx-1). Trx-1 is a ubiquitous protein with redox-active site sequence:-Cys-Gly-Pro-Cys- that is induced by various stressors and Trx-1 inducers, such as X-ray and ultraviolet irradiation, hydrogen peroxide, viral infection, ischemic reperfusion, and nerve growth factor. Trx-1 can protect neurons by scavenging free radicals, by modifying the structure of proteins through the reduction of disulfides bonds and by regulating several transcription factors, NF-kβ, p53, AP-1 and CREB [27,28]. Recent studies have shown that Trx-1 is also involved in drug addiction. In particular, methamphetamine administration increases Trx-1 expression, which in turn was shown to regulate CREB activity [29]. In addition, morphine treatment increased Trx-1 protein levels in nuclear fractions [30]. In the nucleus, Trx-1 might facilitate an interaction between transcription factors, NF-κβ or CREB, with DNA to facilitate transcription of genes [27]. Overall, these findings suggest that Trx-1 might also play an important role in morphine dependence.

Given the possible involvement of Trx-1 in the activation of CREB and the role of CRF as a neuro-regulator in the behavioral and emotional integration of context-specific effects of opioid addiction, in the present study we have assessed: 1) Trx-1 expression, CREB phosphorylation and the co-localization of phospho (p)CREB and Trx-1 in PVN and DG following morphine-induced CPP and 2) the effects of the CRF1R antagonist, CP-154,526, on morphine CPP-induced activation of the CRF system in the PVN and, the DG, as well as the role of CREB phosphorylation and Trx-1 expression in morphine-induced behaviors.

## Results

### Effects of morphine administration in body weight

**Fig 1:**
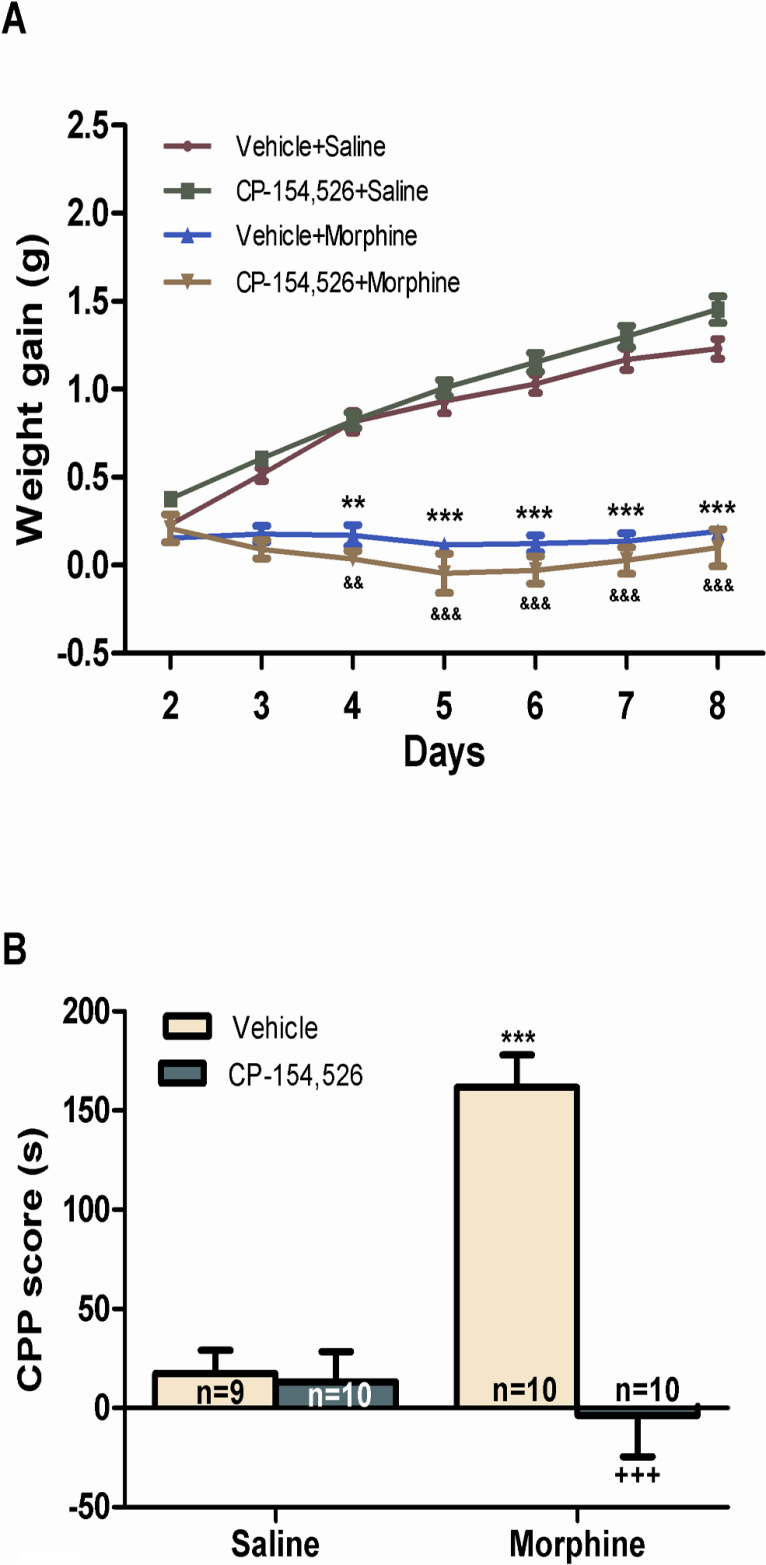
Effects of CP-154,526 on weight gain in mice treated with morphine or saline, n = 11–13 (A). Effect of repeated saline or morphine injection on conditioned place preference, (CPP). CPP induced by morphine administration (6 mg/kg, i.p.) during the conditioning phase (B). The score was calculated for each mouse as the difference between post-conditioning and the preconditioning time spent in the drug-paired compartment. Data are expressed as mean ± SEM. **p<0.01, ***p<0.001 versus the control group treated with vehicle+saline; &&*p*<0.01, &&&*p*<0.001 versus the group treated with CP-154,526+saline; +++*p*<0.001 versus the group treated with vehicle+morphine.

We examined body weight gain from the day 4 of the morphine-administration paradigm (Fig 1A). ANOVA with repeated measures showed significant main effect of time (F_1,46_ = 8.547, *p* = 0.005), morphine treatment (F_1,46_ = 18.927, *p*<0.0001) and a significant interaction between time x morphine treatment (F_1,46_ = 9.169, *p* = 0.004). This indicates that the change in body weight gain depended on the combined influence of time x morphine chronic treatment. The decrease in body weight gain persisted until day 8 in animals treated with vehicle+morphine or CP-154,526+morphine versus their control groups (Fig 1A).

### CP-154,526 inhibited the rewarding effects of morphine

Morphine administration induced a significant place preference to the drug-paired compartment compared to saline groups (Fig 1B). Two-way ANOVA for CPP score revealed a main effect of morphine (F_1,35_ = 14.93, *p* = 0.0005) and CP-154,526 effect (F_1,35_ = 26.47, *p<*0.0001) and a significant interaction between morphine and CP-154,526 effects (F_1,35_ = 23.95, *p<*0.0001). *Post hoc* test showed that the score was significantly (*p*<0.001) higher in mice conditioned by morphine than in the saline-paired group, indicating motivational effects of morphine cues. However, pre-treatment with CP-154,526 significantly (*p*<0.001) decreased morphine-induced CPP (Fig 1B).

### Effects of CP-154,526 on CRF neurons and fibers

**Fig 2:**
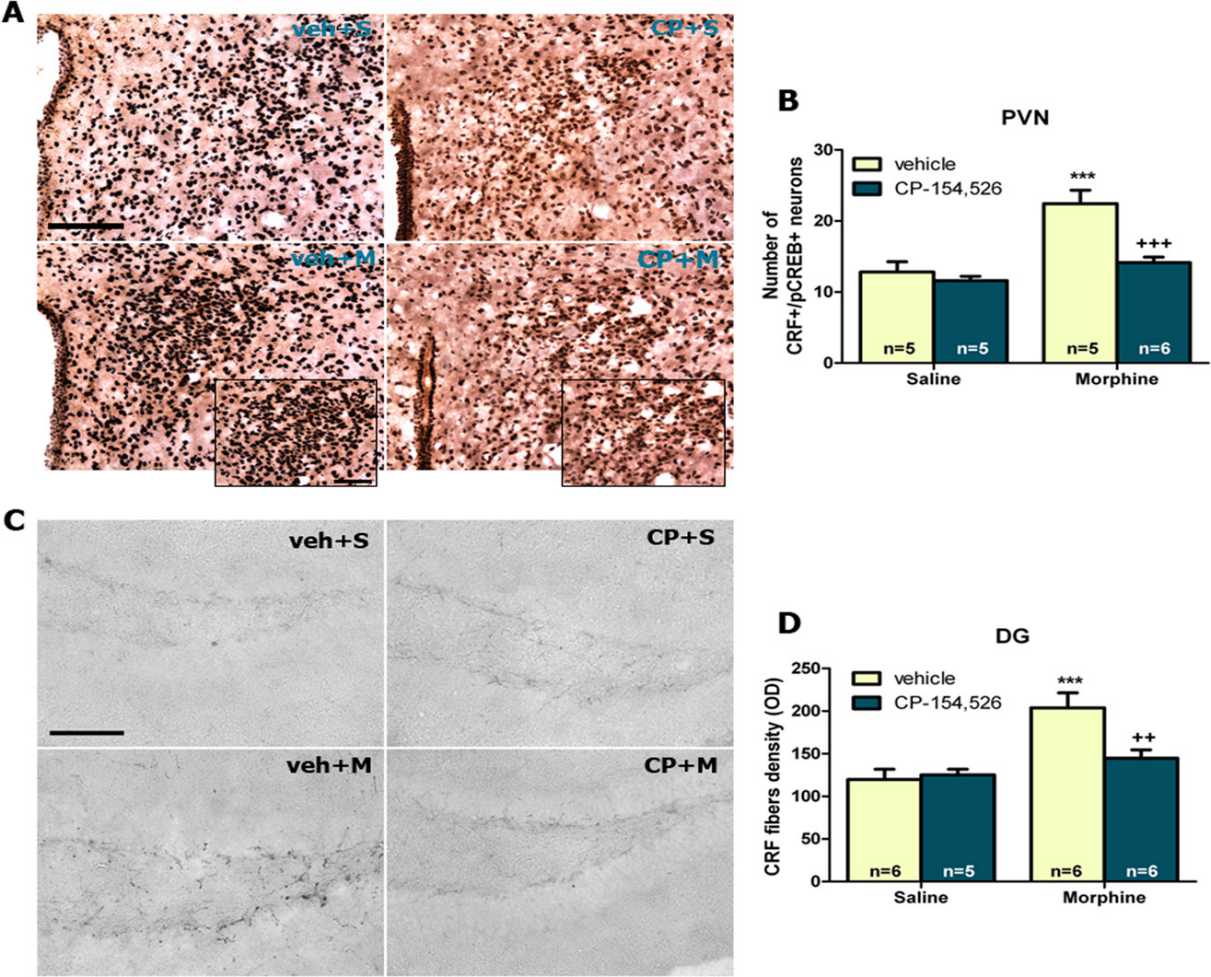
Example of photomicrographs showing CRF/pCREB double-labelling in paraventricular nucleus (PVN) (A). Graph in upper right shows the mean total number or double-labeled neurons (B). Photographs represent immunohistochemical detection of CRF fibers in the dentate gyrus (DG) (C). Graph in downright shows the optical density of CRF immunostaining in the DG (D). Scale bar 100 or 50 μm. Data are expressed as mean ± SEM. ***p<0.001 versus vehicle (veh)+saline (S); ++*p*<0.01, +++p<0.001 versus vehicle+morphine (M). CP-154,526 (CP).

For the analysis of the co-localization of pCREB with CRF-positive neurons in the PVN, two-way ANOVA showed a significant effect of morphine (F_1,17_ = 23.58, *p* = 0.001) and CP-154,526 (F_1,17_ = 14.34, *p* = 0.0015) and also an interaction between morphine and CP-154,526 effects (F_1_,_17_ = 14.34, *p* = 0.0015). Double-labeling experiments showed that pCREB was highly (*p*<0.001) co-localized with CRF-positive neurons in the PVN in morphine-conditioned mice. CP-154,526 pre-treatment decreased the number of CRF/pCREB-positive neurons in morphine-treated mice (Fig 2A and 2B).

We also investigated morphine-induced changes in CRF fibers within DG. Two-way ANOVA showed a main effect of morphine (F_1,19_ = 16.93, *p* = 0.0006) and CP-154,526 effect (F_1,19_ = 4.58, *p* = 0.0456) and an interaction between morphine and CP-154,526 factors (F_1,19_ = 6.57, *p* = 0.0190). *Post hoc* analysis showed a significant increase of CRF-immunoreactive fibers after morphine-CPP training compared to controls (p<0.001; Fig 2D). Pre-treatment with CP-154,526 attenuated the enhancement in CRF-immunoreactive fibers in DG induced by morphine (Fig 2C and 2D).

### Effects of CP-154,526 on CREB activity in PVN and DG after morphine-induced CPP

**Fig 3:**
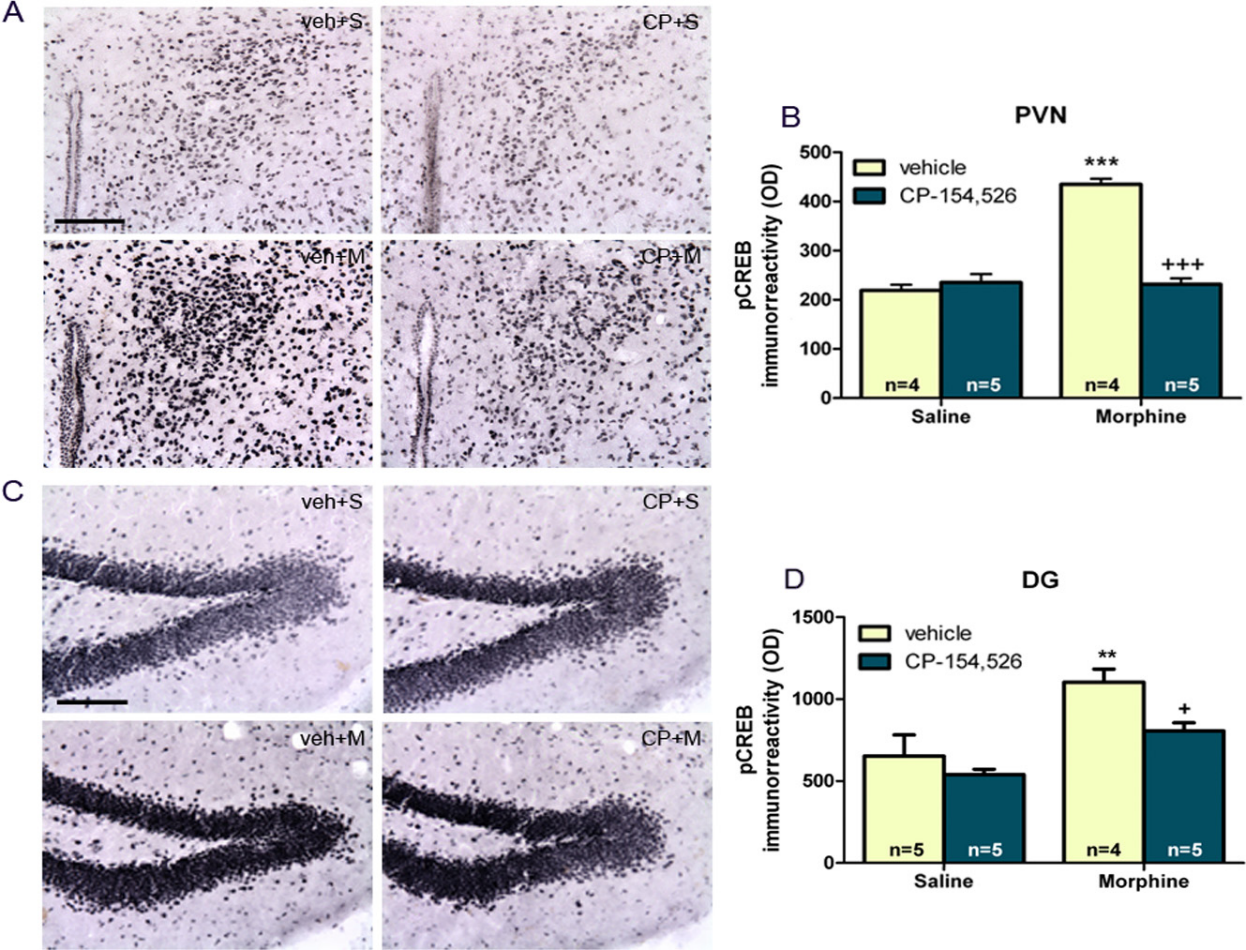
CREB activation in morphine CPP mice. Photographs represent immunohistochemical detection of pCREB in paraventricular nucleus (PVN) (A), and dentate gyrus (DG) (C). Scale bar 100 μm. Quantitative analysis of pCREB immunohistochemistry in PVN (B) and DG (D). Data are expressed as mean ± SEM. **p<0.01, ***p<0.001 versus vehicle (veh)+ saline (S); +*p*<0.05, +++*p*<0.001 versus vehicle+morphine (M). CP-154,526 (CP).

With reference to hypothalamic PVN (Fig 3A and 3B), two-way ANOVA for pCREB revealed a main effect of morphine (F_1,14_ = 60.70, *p*<0.0001) and CP-154,526 (F_1,14_ = 47.12, *p<*0.0001) and an interaction between morphine and CP-154,526 factors (F_1,14_ = 65.13, *p<*0.0001). Tukey *post hoc* test showed that there was a significant enhancement in pCREB expression in the morphine-treated groups compared to controls. This increase in pCREB expression was attenuated by CP-154,526 pre-treatment (Fig 3A and 3B). At DG level (Fig 3C and 3D), two-way ANOVA revealed a main effect of morphine and CP-154,526 (F_1,15_ = 18.85, *p* = 0.0006; F_1,15_ = 6.15, *p* = 0.0255, respectively). No significant morphine x CP-154,526 interaction effect was observed (F_1,15_ = 1.25, *p* = 0.2819). *Post hoc* test revealed an increase in pCREB immunoreactivity in morphine-injected mice (Fig 3D) and this effect was attenuated by CP-154,526 pre-treatment (Fig 3C and 3D).

### Trx-1 expression after morphine-induced CPP

**Fig 4:**
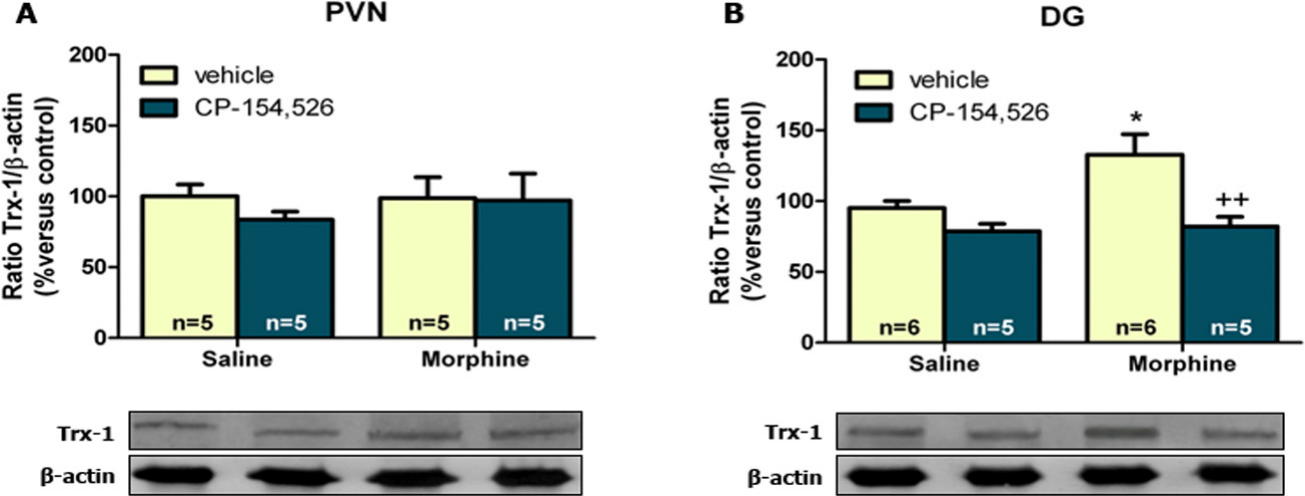
Western-blotting analysis of Trx-1 in paraventricular nucleus (PVN) (A) and dentate gyrus (DG) (B). Each bar represents the mean optical density ± SEM; values are expressed as % of controls.*p<0.05 versus vehicle+saline; ++*p*<0.01 versus vehicle+morphine.

Since CREB can be activated by Trx-1 [27], we have evaluated the expression of Trx-1 in PVN (Fig 4A) and DG (Fig 4B). Two-way ANOVA for Trx-1 expression in PVN revealed no significant effects of morphine (F_1,16_ = 0.22, *p* = 0.6466) or CP-154,526 (F_1,16_ = 0.47, *p* = 0.5021) or an interaction between these factors (F_1,16_ = 0.32, *p* = 0.5773) (Fig 4A).

Analysis of the expression of Trx-1 in DG by a two-way ANOVA test, revealed a significant effect of morphine (F_1,19_ = 0.0362, p = 0.0362) and CP-154,526 pre-treatment (F_1,16_ = 13.47, *p* = 0.0016) but not a significant interaction between morphine and CP-154,526 (F_1,19_ = 3.54, *p* = 0.0754). *Post hoc* test revealed a significant (p<0.05) increase in Trx-1 expression in the morphine-induced CPP group compared to controls, that was significantly reduced by CP-154,526 administration (Fig 4B).

### Number of pCREB and Trx-1 positive neurons after morphine-induced CPP

**Fig 5:**
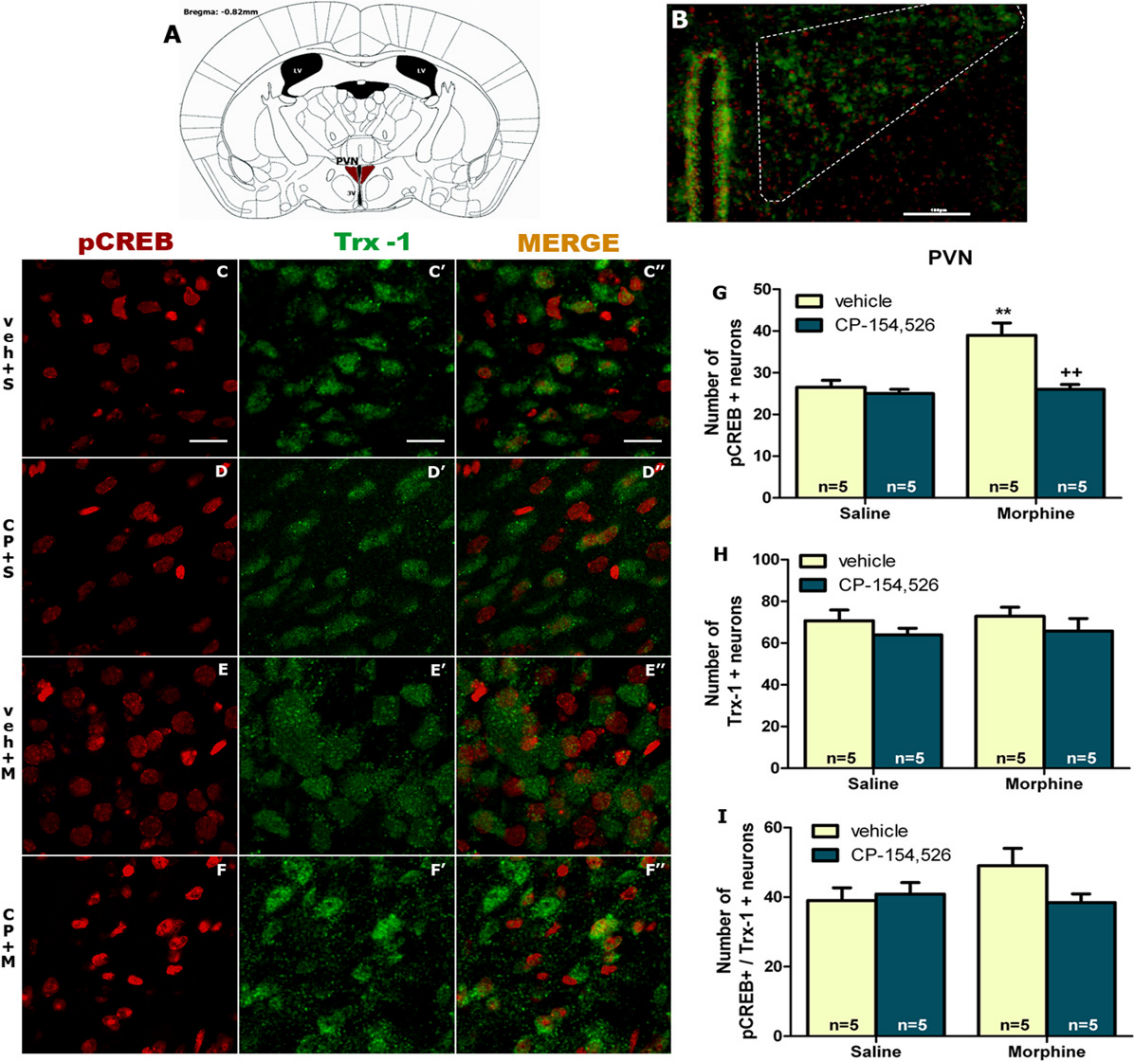
The analyzed region within paraventricular nucleus (PVN) is schematically illustrated in A (diagram from Franklin & Paxinos 2008); coordinate -0.82 mm from Bregma. (B) Example of magnification micrograph showing a midbrain coronal section of mice immunostained for pCREB and Trx-1; scale bar 100 μm. Representative confocal images of pCREB (reed) (C-F) and Trx-1 (green) (C’-F’). Merged images are shown in C”-F” (pCREB/Trx-1). Scale bars 20 μm. Graphs in right show the mean total number of pCREB (G) or Trx-1 (H) neurons and the double-labelled neurons (pCREB/Trx-1) (I). Data are expressed as mean ± SEM. **p<0.01, versus vehicle (veh)+ saline (S); ++*p*<0.01 versus vehicle+morphine (M). CP-154,526 (CP).

To assess the capability of morphine conditioning to induce CREB phosphorylation through Trx-1 signaling in PVN and DG, a double-labeled immunofluorescence for pCREB and Trx-1 was performed. In PVN, two way ANOVA for pCREB positive neurons revealed a significant main effect of morphine (F_1,16_ = 13,06 *p* = 0.023) or CP-154,526 effect (F_1,16_ = 15,03 *p* = 0.0013) as well as morphine x CP-154,526 treatment interaction (F_1,16_ = 9,44, *p* = 0.0073). However, two-way ANOVA for Trx-1- positive neurons showed no significant effect of morphine (F_1,16_ = 0,17, *p* = 0.6833) or CP-154526 (F_1,16_ = 2,05, *p* = 0.1712) or interaction (F_1,16_ = 0,00, *p* = 0.9769). Tukey *post hoc* test showed that morphine-induced CPP significantly (*p*<0.01) increased the number of pCREB-positive neurons compared with saline-treated groups. However, CP-154,526 pre-treatment induced a significant (*p*<0.01) decrease in the number of pCREB-positive neurons (Fig 5G). CP-154,526 pre-treatment did not induce any significant change in the number of Trx-1 positive neurons (Fig 5H).

Two-way ANOVA for pCREB/Trx-1 co-localization revealed no effect of morphine (F_1,16_ = 0.17, *p* = 0.6833) or CP-154,526 (F_1,16_ = 2,05 *p* = 0.1712) or morphine x CP-154526 interaction (F_1,16_ = 0,00 *p* = 0.9769) (Fig 5I, 5C´´, 5D´´, 5E´´, and 5F´´).

**Fig 6:**
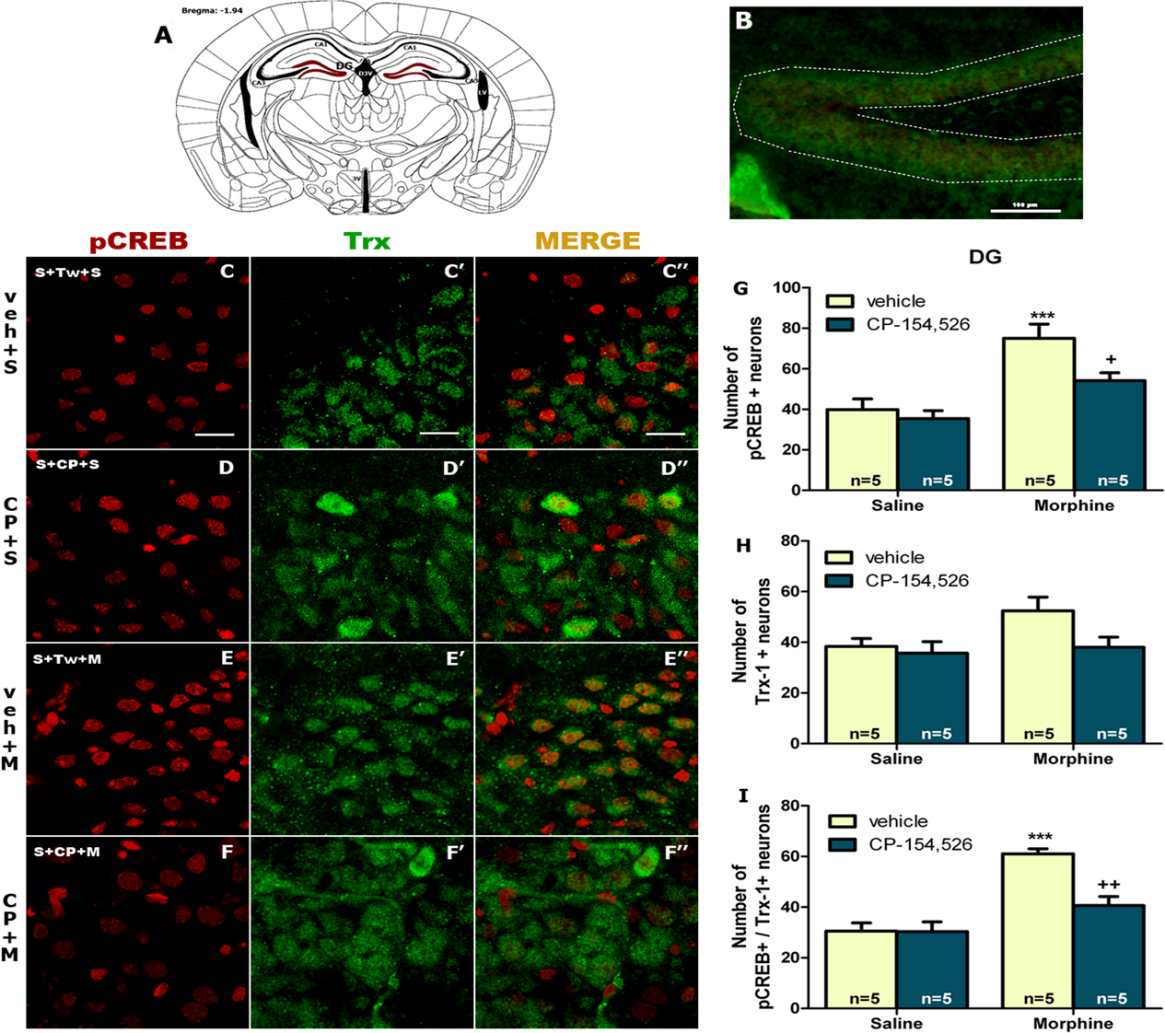
The analyzed region within dentate gyrus (DG) is schematically illustrated in A (diagram from Franklin & Paxinos 2008); coordinate -1.94 mm from Bregma. (B) Example of magnification micrograph showing a midbrain coronal section of mice immunostained for pCREB and Trx-1; scale bar 100 μm. Representative confocal images of pCREB (red) (C-F) and Trx-1 (green) (C’-F’). Merged images are shown in C”-F” (pCREB/Trx-1). Colocalization is shown by yellow-orange neurons in the merged images. Scale bars 20 μm. ***p<0.001, versus vehicle (veh)+saline (S); +*p*<0.05, ++*p*<0.01 versus vehicle+morphine (M). CP-154,526 (CP).

Regarding the analysis of the number of pCREB-positive neurons in DG, two-way ANOVA revealed a main effect of morphine (F_1,16_ = 26.86, *p<*0.0001) or CP-154,526 effect (F_1,16_ = 5.94, *p* = 0.0268) with no significant interaction between morphine and CP-154526 treatment (F_1,16_ = 2.53, *p* = 0.1311). As shown in Fig 6G, morphine-induced CPP significantly (*p*<0.001) increased the number of pCREB-positive neurons in DG and CP-154526 pre-treatment significantly blocked (*p*<0.05) this enhancement. However, two-way ANOVA for the number of Trx-1 positive neurons revealed no significant effects of morphine (F_1,16_ = 3.55, *p* = 0.0779), CP-154,526 (F_1,16_ = 3.88, *p* = 0.0665), or interaction between morphine x CP-154,526 factors (F_1,16_ = 1.84, *p* = 0.1936) (Fig 6H).

For assessing pCREB/Trx-1 co-localization in DG, two-way ANOVA showed significant main effects of morphine (F_1,16_ = 40.54, *p*<0.0001), CP-154,526 (F_1,16_ = 10.38, *p* = 0.0053) and a significant interaction between morphine and CP-154,526 treatment (F_1,16_ = 10.02, *p* = 0.0060). *Post hoc* test revealed an increase in the number of pCREB positive/Trx-1 positive neurons. CP-154,526 treatment decreased the number of pCREB neurons that express Trx-1 (Fig 6I, 6C´´, 6D´´, 6E´´and 6F´´).

## Discussion

In accordance with previous findings [31–34], present study demonstrated that morphine administration produced a significant CPP. The acquisition of morphine-induced CPP was abolished by pretreatment with CP-154,526 suggesting a key role of CRF1R in the rewarding effects of morphine. The CRF1R antagonist was administered by systemic via since it is a non-peptide antagonist that crosses the blood brain barrier and reaches maximal brain concentration after 20 min [35]. In addition, there are many studies in the literature using systemic CP-154,526 administration [36–40].

According to the results of the present study previous data demonstrated that CP-154,526 but not antisauvagine-30 (a selective CRF2 receptor antagonist) administration prevented cocaine-induced CPP [41]. Altogether these results suggest that CRF signaling, through CRF1R, is essential for the acquisition of CPP induced by opioid drugs.

Hippocampus is a brain region known to participate in associative processes such as declarative memory. This area has direct excitatory inputs to the NAc and can activate dopaminergic neurons of the VTA [42]. Recently, it has been suggested that PVN response to different stimulus is reinforced through indirect stimulation of mesolimbic dopamine neurons of VTA [43]. In addition, it has been suggested that PVN may have a role in the reinforcing effects of opioids [43]. We have chosen DG to perform our experiments because understanding how the formation of drug-reward memories alters the neurobiology of the hippocampal DG may shed light on the later and more persistent aspects of addiction, We have compared this area with PVN for two reasons: 1) The implication of this structure in the regulation of the reinforcing properties of drugs and drug-associated cues is less known than structures involved in the reward circuitry; 2) PVN and hippocampus are related with mesolimbic pathways; and 3) PVN is an stress area and it is known that stress can modify memory processes. Altogether, these data indicate a role of both intra-hypothalamic (PVN) and hippocampal (DG) brain areas in the rewarding effects induced by drugs of abuse.

Although many studies have implicated CRF signaling in the anxiogenic-like and aversive motivational effects of drug withdrawal [44], its role in mediating the rewarding effects of opiates remains unclear. CRF-immunoreactive fibers densely innervate many intrahypothalamic and extrahypothalamic brain areas, including hippocampus. Moreover, CRF, via CRF1R, enhances neuronal activity propagation from the classical hippocampal input region, DG, to the hippocampal area CA1 [45]. CRF is expressed in hippocampal interneurons within the pyramidal cell layer [18]. The supramamillary (SuM) nucleus of the hypothalamus is thought to serve as an interface relaying input to hypothalamic and limbic structures involved in the control of behavioral functions [46]. SuM region is relatively unique among hypothalamic structures in which it sends a large, direct projection to DG [47].

CREB is important in the switch from short-term to long-term memory, and plays a central role in the formation of long-term memory. Drug addiction and learning and memory share certain intracellular signaling cascades which involve the activation of the transcription factor CREB [48]. In agreement with previous studies [42,49], present study demonstrated that the number of pCREB positive neurons in PVN and DG was increased significantly after morphine CPP expression. Since CRF1R is coupled to stimulatory G protein Gαs and can thus activate PKA and subsequently CREB [23], in the present study we have determined whether CRF1R signaling is important in mediating CREB activity after morphine-induced CPP. Administration of the CRF1R antagonist, CP-154,526, abolished morphine-induced enhancement of pCREB positive neurons, totally in PVN and less extend in DG. CREB involvement in morphine dependence has been previously supported by studies showing that CREB knockout mice do not exhibit morphine-induced CPP [50], suggesting that CREB function is necessary for the rewarding properties of morphine.

CRF system has been shown to be involved in the modulation of the anxiolytic effects of environmental enrichment [51] and in the stress-induced cocaine CPP [52]. In the present study, we showed that most of the CRF positive neurons in PVN co-express pCREB in morphine-conditioned mice. Moreover, we observed an increase in the optical density of CRF fibers in DG following morphine treatment. These changes were blocked by the administration of a CRF1R antagonist. In the classical pathway, CRF binding to CRF1R leads to signal transduction across the cell membrane resulting in activation of heterotrimeric G-proteins. The primary target is represented by the activation of Gαs (AMPc/PKA/CREB). However, CRF through CRF1R can activate other G-proteins such as Gαq [inositol triphosphate (IP3)]. Increase in the concentration of secondary messengers (cAMP, IP3 and Ca2+) in cells triggered by CRF1R ligands, results in activation of multiple transcriptional factors including CREB, AP-1, NF-κB, and the calcium response element (CARE) [53–59]. In this regard, CP-154,526, by blocking the postsynaptic CRF1R, inhibited CREB phosphorylation in PVN and DG. In addition, morphine induced an increase in optical density in CRF fibers in DG, suggesting an increased CRF release. This increase was prevented by pre-treatment with the CRF1R antagonist. Since the activation of CRF1R increases Ca2+ levels, it is possible that CP-154,526 inhibits CRF release by blocking pre-synaptic CRF1R in PVN.

Previous studies suggest that CREB phosphorylation is a convergence point for multiple kinases and acts as a molecular switch for controlling gene activation kinetics [42]. CREB activity can also be regulated by the family of redox-protein, Trx-1 [27]. In addition to its antioxidant activity, Trx-1 plays an important role in cellular signaling by regulating various components of the signal transduction pathway, such as nuclear factor-κB, p38 mitogen-activated protein kinases, activator protein-1, CREB, estrogen receptor, glucocorticoid receptor and p53 [60]. Present study is the first to show that morphine-induced CPP increased Trx-1 expression in DG. Trx-1 might activate CREB phosphorylation, thus increasing the rewarding effects of morphine; however further studies are needed in order to fully elucidate the cellular mechanisms underlying this regulation. In line with our finding, increased Trx-1 expression has been also observed after morphine or metamphetamine administration [29,30]. The increased activity of CREB by methanphetamine was suppressed by Trx-1siRNA, suggesting that Trx-1 is essential for CREB activation [29]. In addition, morphine-induced increase in Trx-1 expression was blocked by naloxone, demonstrating that this effect is regulated by the activation of opioid receptors [30]. Our finding indicating a positive association between the rewarding effects of morphine and Trx-1 expression is in contrast with a previous study [61] that shows that geranylgeranylacetone induces Trx-1 expression in the NAc and concomitantly attenuates morphine-induced CPP. These discrepancies might be explained by the differential modulation roles of NAc and hippocampus. Similarly, CREB expression was increased in hippocampus but decreased in NAc [42] following morphine conditioning, suggesting differential regulation of CREB activation in different brain areas.

A large number of pCREB/Trx-1 double-labeled neurons were observed in DG. The presence of Trx-1 in pCREB positive neurons in DG suggests that CREB may be activated by Trx-1 in DG, a brain region involved in memory consolidation. Considering the important role of Trx-1 in maintaining the cellular reducing environment, the up-regulation of Trx-1 expression after morphine-induced CPP might be associated with a compensatory mechanism of stress systems for the maintenance of neuroprotection. Further studies are needed to investigate this question.

Given the evidence for the promiscuity of CRF coupling to intracellular signaling pathways, and the profound influence of CRF on CREB phosphorylation, our study aimed to determine the specific signaling pathway by which Trx-1-induced CREB phosphorylation in DG. Using a CRF1R antagonist, we showed that morphine-induced CPP produces an increase in Trx-1 expression in DG which was completely blocked by pre-treatment with CP-154,526. We also showed that morphine-induced CPP produced an increase in the number of pCREB neurons co-expressing Trx-1, suggesting a role for CRF1R in CREB phosphorylation, possibly via a Trx-1 dependent mechanism. The exact mechanism by which the CRF system regulates Trx-1 signaling in DG is not fully understood. One hypothesis is that pCREB might bind to CRE in the 5’-upstream sequence of Trx-1 gene and induce Trx-1 expression to regulate CREB phosphorylation. Supporting this hypothesis, it has been demonstrated that ephedrine induces Trx-1 expression through the β-adrenergic receptor/cyclic AMP/PKA/DARPP-32 signaling pathway [62]. In addition, methamphetamine-induced Trx-1 expression and CREB activity in rat pheochromocytoma cells was shown to be regulated by Trx-1 [29].

Present study cannot rule out that the changes observed after morphine conditioning could be due to morphine exposure, or drug-associated context. However, previous studies from our laboratory [34] clearly demonstrated that exposure to a drug-associated context leads to CRF activation. Recently, it has been suggested that morphine only alter hippocampal function when paired with the place conditioning stimulus, suggesting that this structure modulates not the primary reinforcing stimulus produced by morphine, but rather the association of this stimulus with the paired contextual environment [63]. Therefore, it is likely that the environmental cues associated with drug administration play a critical role in the adaptive changes observed in PVN and DG, brain areas involved in reward pathways.

In summary, the findings of the present study indicate that morphine-induced CPP increases Trx-1 expression and the number of pCREB positive neurons in DG via CRF1R activation. These data point out the role of CRF1R in the adaptive changes observed after morphine-induced CPP and could contribute to establish novel molecular targets for modulating the adverse effects of morphine dependence.

## Materials and Methods

### Animals

Adult male Swiss mice (Harlan, Barcelona, Spain) weighing 25–30 g at the beginning of the experiments were housed five to seven per cage in a temperature-and humidity-controlled environment with a constant 12-h light/dark cycle (lights on at 8 am). Mice were habituated to the testing room for at least 1 week prior to experimental start and they were handled daily during this week to minimize stress. Access to water and food was available *ad libitum*. All animal experiments were carried out in accordance with the European Communities Council Directive of 24 September 2010 (2010/63/UE) and were approved by the local Committees for animal research (Comité Ético de Experimentación Animal, CEEA; Universidad de Murcia; RD 53/2013). Protocols were designed to minimize the number of experimental animals and to minimize their suffering.

### Conditioned place preference

**Fig 7:**
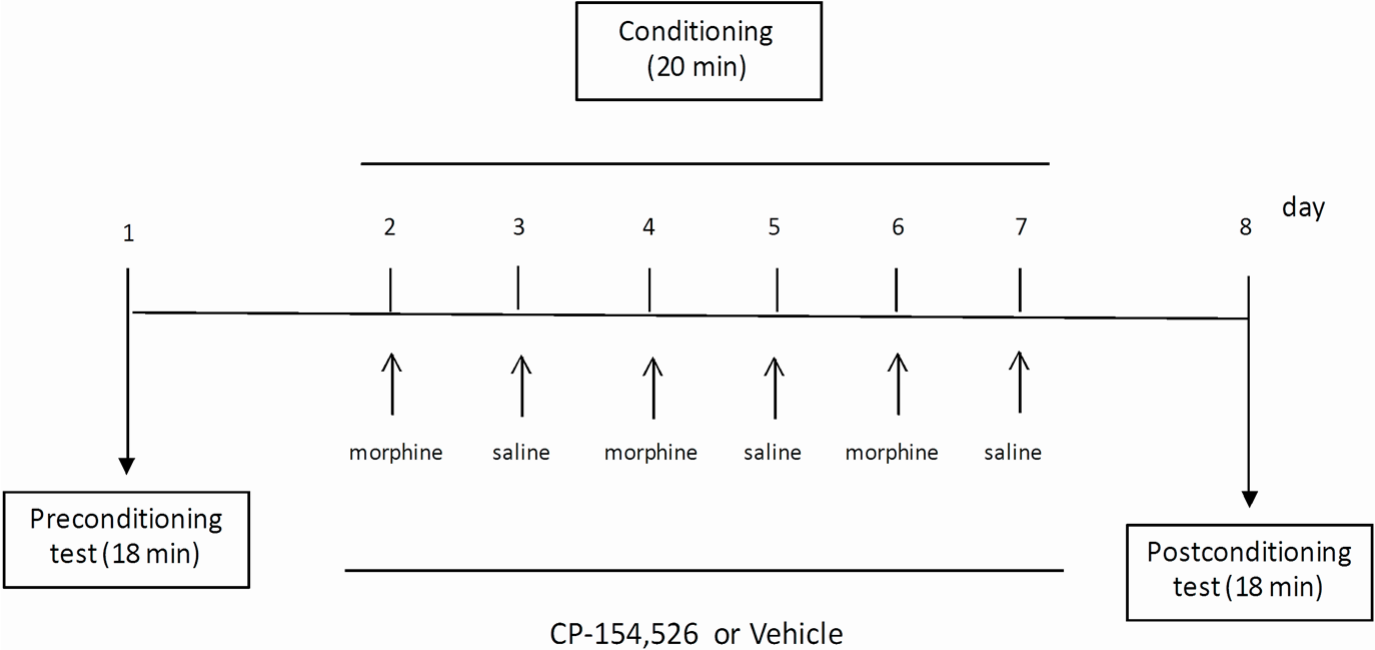
Experimental schedule for the conditioned place preference training. Mice were pre-treated with CP-154,526 (30 mg/kg, i.p.) or vehicle (Tween 80) 30 min before saline or morphine for 6 days in the conditioning period.

Mice were conditioned and tested during the light cycle phase in the CPP apparatus as described previously [16]. CPP procedure consisted of three different phases: one preconditioning, six conditioning and one post-conditioning. On day 1 (pre-conditioning test), mice explored freely the entire apparatus for 18 min. The time spent in each compartment was recorded, and the animals that spent less than 390 s in either chamber were considered not to be neutral in preference for either side and were excluded from future analysis (n = 3). After the pre-conditioning test, animals were assigned randomly to one of the experimental groups using a counterbalanced design: 1) vehicle+saline, 2) CP-154,526+saline, 3) vehicle+morphine and 4) CP-154,526+morphine. During the conditioning phase, mice received morphine (6 mg/kg i.p.) on days 2, 4 and 6 and saline on days 3, 5 and 7. Control animals received saline injection every single day. All mice were pretreated with vehicle (Tween-80 10%, i.p.) or CP-154,526 [*N*-butyl-*N*-ethyl-2,5-dimethyl-7-(2,4,6-trimethyl-phenyl)pyrrolo(3,2-*e*)pyrimidin-4-amine](30 mg/kg i.p.), 30 min before morphine or saline injection during the conditioning phase (Fig 7). The doses of CP-154,526, a selective CRF1R antagonist, were selected based on previous studies from our laboratory [64,65]. Immediately after saline or morphine injections, animals were placed in the CPP compartment assigned to each animal for 20 min. Conditioning was conducted as previously described in detail using an unbiased procedure [66]. During the post-conditioning tests (day 8), animals were allowed to explore both CPP compartments for 18 min. Acquisition of place conditioning was defined as a statistically significant increase in time spent in the drug-paired compartment during the post-conditioning test session compared to pre-conditioning test.

### pCREB immunohistochemistry

Mice were deeply anaesthetized with a subletal dose of pentobarbital after the end of the post-conditioning test session and perfused transcardially with an ice-cold fixative solution containing 4% paraformaldehyde in phosphate buffer (0.1 M) pH 7.4. Then, brains were removed and fixed in a solution containing sucrose (30%) for a period of 3 h. After that, brains were placed in phosphate buffered saline (PBS) containing 30% sucrose overnight at 4°C. Series of 30-μm coronal brain sections from PVN (bregma: -0.70mm) and DG (bregma: -1.82mm) [67] were collected and incubated with a rabbit anti-pCREB [1:750 in normal goat serum (NGS)-PBS; Upstate] for 3 days at 4°C. This was followed by application of a biotinylated anti-rabbit IgG (diluted 1:200 for 1 h) in NGS-PBS (Vector), and then 1 h with the avidin-biotin complex at room temperature. Visualization of the antigen-antibody reaction sites was performed using 0.033% 3,3-diaminobenzidine (DAB; Sigma Chemical Co.) intensified with nickel and 0.014% H_2_O_2_ in 0.05 M Tris-HCl buffer for 7 min. The reaction was terminated by rinsing in PBS. Sections were mounted on gelatine-coated slides, dehydrated through graded concentrations of alcohols, followed by a xylene-washing step and coverslipped with dibutylphtalate (DPX).

### Double-labeling immunohistochemistry of pCREB-immunoreactive nuclei and CRF positive neurons

For determining pCREB-immunoreactivity, tissue sections from each mouse in each treatment group were processed using DAB nickel intensification. CRF was then visualized using DAB chromogen only. Briefly, pCREB immunohistochemistry was performed as described previously (dilution of primary antibody: 1:500), and pCREB antibody-peroxidase complex was visualized by using a mixture of NiSO_4_.6H2O (33.2 mg/mL), DAB (0.033%) and 0.014% H_2_O_2_ in 0.175 M sodium acetate solution, pH 7.5. Sections were then incubated with an anti-CRF antibody (diluted 1:500) for 72 h at 4°C. A biotinylated anti-rabbit IgG (diluted 1:200 for 1 h) was used as a secondary antibody. The CRF antibody-peroxidase complex was stained in 0.033% DAB and 0.014% H_2_O_2_ in 0.05 M Tris-HCl buffer.

### Immunofluorescence study

For pCREB and Trx-1 immunofluorescence, brain sections containing the PVN and DG were pre-treated with 10 mM citrate buffer, pH 6, for 30 min at 65°C in order to increase antigen retrieval and penetration of the antibodies into the tissues. Sections were blocked with 1% Triton X-100 for 5 min and with 2% horse serum in 0.1 M PBS, pH 7.4, for 60 min. Sections were incubated with combined primary antibodies raised in different species; mouse anti-pCREB (Ser133) (1:750 dilution; Millipore) and rabbit anti-Trx-1 (1:1000 dilution; Abcam) for 48 h at room temperature. For multiple staining, incubation with primary antibodies was followed by 4 h incubation with the secondary antibodies [Alexa 488 and Alexa 594 (1:1,000 dilution; Invitrogen, Molecular Probes)]. Sections were washed six times in PBS and then mounted on slides. Slides were coded and randomised prior to quantitative analysis.

### Immunofluorescence image analysis

Inmunofluorescent images were captured (x20 objective) using Leica SCN400F slide scanner and they were then displayed on a computer screen where Leica SCN400F client. Digital Hub 3.0 application software was used for standardised images by setting the exposure and brightness to a constant value. The images were then saved in JPEG format, and analysed using Image J-64 software. On each section, 3–4 circles (59.2 mm diameter) 200 mm apart were used to analyze the DG or the PVN. Results are given as number of cells per section.

Images were obtained using a Leica DM6000 Confocal Microscope (Leica Microsystems CMS GmbH, Mannheim, Germany) using 488-nm excitation for Alexa Fluor 488 and 543-nm excitation for Alexa Fluor 594. Emitted light was detected in the range of 515–530 nm for Alexa Fluor 488 and 605 nm for Alexa Fluor 594. Every channel was captured separately to avoid spectral cross talking. Images were deconvolved using Huygens Essential 3.6 by Scientific Volume Imaging.

### Quantification of CRF fibers and (p)CREB immunoreactivity

CRF fibers in DG as well as pCREB immunostaining in the PVN and DG were quantified bilaterally by an observer who was blind to the treatment groups. The density of pCREB-like immunoreactivity was determined using a computer assisted image analysis system (QWIN, Leica, Madrid, Spain). This system consists of a light microscope (DM4000; Leica) connected to a video camera (DFC290, Leica) and the image analysis computer. CRF fibers were analyzed by measuring the optical density with a computer analyzer (ScionImage) as previously it has been described [68]. A square field (195x195 μm) was superimposed upon the captured image (x20 magnification) to use as reference area.

### Quantification of pCREB-positive/CRF-positive neurons

pCREB-positive CRF neurons were identified as cells with a marked brown cytosolic deposits for CRF-positive staining and blue/dark nuclear staining for pCREB. A square field (195x195 μm) was superimposed to be used as a reference area. Double-labeled pCREB neurons were counted in four to five sections from each animal in the PVN and DG bilaterally. The CRF- positive cells without a visible nucleus (pCREB negative CRF cells) were also included in the analysis.

### Western Blotting

Mice were decapitated 1 h after the end of the post-conditioning test session. PVN and DG were micro-punched from frozen brain sections (500 μm), sectioned using a cryostat, according to the mice brain atlas of Frankin and Paxinos [67]. All micro-punched samples were stored frozen at -80°C until use. PVN and DG were then placed in a buffer containing phosphate buffered saline, 10% sodium dodecyl sulfate (SDS), protease inhibitors and a phosphatase inhibitor Cocktail Set, homogenized and sonicated for 30 s before centrifugation at 6.000 g for 10 min at 4°C. Samples containing 20 μg of protein were loaded on a 10% SDS/polyacrylamide gel, electrophoresed and transferred onto polyvinylidene difluoride membranes (Millipore, Bedford, MA, USA). Nonspecific binding of antibodies was prevented by incubating the membranes in 1% bovine serum albumin (BSA) in Tris-buffered saline Tween-20 (TBST; 10 mM Tris HCl, pH 7.6, 150 mM NaCl, 0.15% Tween 20). The blots were incubated overnight with the following primary antibodies: rabbit polyclonal anti-Trx (1:1000 dilution; Abcam) in TBST with BSA at room temperature. After several washing steps in TBST, membranes were incubated with peroxidase-labeled secondary antibodies (anti-rabbit sc-2004 at 1:5000) for 1 h at room temperature. After washing, immunoreactivity was detected with an enhanced chemiluminescent/chemifluorescent western blot detection system (ECL Plus, GE Healthcare, UK) and visualized by a Typhoon 9410 variable mode Imager (GE Healthcare). We used β-actin as the loading control for all the experiments. Blots were subsequently re-blocked and probed with rabbit polyclonal anti-β-actin (1:1000; Cell Signaling Technology Inc., Danvers, MA, USA). The ratio of Trx-1 / β-actin was plotted and analyzed. Protein levels were corrected for individual levels.

### Drugs and Reagents

Morphine hydrochloride was obtained from Alcaliber Labs (Madrid, Spain), dissolved in sterile 0.9% saline and injected interperitoneally (i.p.) in a volume of 0.1 ml/10 g of body weight. Reagents used were: protease inhibitors (Roche Diagnostics, Indianapolis, IN); phosphatase inhibitor cocktail set (Calbiochem, San Diego, CA); goat and horse serum (Sigma-Aldrich); avidin-biotin complex (Vector Laboratories, Burlingame, CA); and nickel sulfate (Sigma-Aldrich). CP-154,526, kindly provided by Pfizer (New York, NY), was dissolved in 10% Tween 80 (Sigma-Aldrich).

### Statistics

Data are expressed as mean±SEM. Analysis performed using two-way ANOVA followed by a *Tukey post hoc* test. Student’s t-test was used when comparison were restricted to two experimental groups. Differences with a *p*-value < 0.05 were considered significant.
